# Prevalence of household food insecurity and its association with food waste

**DOI:** 10.1186/s42506-024-00168-6

**Published:** 2024-09-03

**Authors:** Rian Diana, Drajat Martianto, Yayuk F. Baliwati, Dadang Sukandar, Agung Hendriadi

**Affiliations:** 1 Department of Nutrition, Faculty of Public Health, Universitas Airlangga, Surabaya, Indonesia; 2 Program of Nutrition Science, Graduate School, IPB University, Bogor, Indonesia; 3 Department of Community Nutrition, Faculty of Human Ecology, IPB University, Bogor, Indonesia; 4 Agroindustry Research Center, National Research and Innovation Agency, Jakarta, Indonesia

**Keywords:** Diary method, FIES, Food access, Food expenditure, Food security, Food waste, Household, Waste composition analysis

## Abstract

**Background:**

Household food waste significantly contributes to overall food waste. While the relationship between food security and food waste has been extensively studied at the macro level, there is a need for research focusing on the quantitative association between food security and food waste at the household level in developing countries, particularly in Southeast Asia. This study aims to estimate the prevalence of household food insecurity and household food waste and to examine the association between household food security and food waste using direct measurements.

**Subjects and methods:**

A total of 215 households in Bogor Regency, Indonesia, participated in this cross-sectional study. Food waste was measured using waste composition analysis and a 7-day diary. The Food Insecurity Experience Scale (FIES) questionnaire was used to assess household food security, while household income and the proportion of food expenditure were considered confounding factors. The association between food security and food waste was examined using Kendall tau-b and ordinal logistic regression.

**Results:**

The prevalence of household food insecurity was 18.6%, and the average household food waste was 77 kg/cap/year. Cereals, tubers, and their derivatives (especially rice) and vegetables were major contributors to edible waste, while fruits dominated inedible waste. A negative association was observed between food waste and household food security (edible FW: *p* = 0.044, *r* = -0.110; total FW: *p* = 0.038, *r* = -0.114), suggesting that household food waste decreases as the severity of food insecurity increases. However, after adjusting for household income, the proportion of food expenditure, and the education levels of spouses, this association became statistically insignificant (*p* > 0.05).

**Conclusion:**

There was no significant association between household food security status and food waste. Household income plays a significant role in determining the quantity of household food waste, as higher income is associated with increased food waste. Strategies to prevent and reduce food waste should focus on major contributors such as rice and vegetables, especially among families with higher food accessibility.

## Introduction

Food waste (FW) is a critical issue and has become a global concern. In 2019, the global average FW per capita was 121 kg/year, with households generating 61% of it [1]. Urban households generate more FW than their rural counterparts [2, 3]. High levels of FW have a significant impact on food system sustainability across three dimensions: economic, environmental, and social, including food security and nutrition at the micro, meso, and macro levels [4].

The paradoxical global situation of high FW amidst a large number of people experiencing hunger and food insecurity is staggering. In 2019, approximately 25.9%, or 2 billion, of the world's population experienced moderate to severe food insecurity, indicating that they cannot access sufficient and nutritious food, despite not necessarily being hungry [5]. Reducing FW can play a significant role in improving sustainable food security and reducing global hunger [6]. Food loss and waste are major barriers to achieving sustainable food and nutrition security [4].

The association between food security and food waste at the macro level has been extensively discussed [4, 7]. Meanwhile, at the household level, reducing food waste can significantly improve food security, especially in households where food availability is a significant issue [8]. Food waste can also lead to a reduction in food availability in terms of mass, calories, and nutrients [4, 8]. However, the quantitative association between food security and food waste at the household level needs further investigation, especially in scenarios where food availability is not a major concern.

Few studies have explored the association between food waste and food security at the household level. Studies in Saudi Arabia have revealed conflicting results compared to those in Iran and the USA [9–11]. However, these studies relied on indirect measurements to assess household FW. At the household level, FW can lead to a decrease in food consumption and an increase in food expenditures [4]. FW can also lead to a reduction in food availability in terms of mass, calories, and nutrients [4, 8].

Limited studies have been conducted on the direct measurement [12] and comparison of the quantity of FW between food-secure and food-insecure households at the household level [11]. Further research is needed in this area [10]. Notably, no quantitative study has been conducted on the association between FW (especially using direct measurements) and household food security in developing countries, such as those in Southeast Asia. This study aims to address this gap by analyzing the association between FW and household food security.

## Methods

### Study design and population

This cross-sectional study was conducted in Bogor Regency, Indonesia, from September to October 2022. The 2020 Indonesian Population Census revealed that Java Island holds the highest population share in Indonesia at 56.1%, with nearly one-third (31.8%) residing in West Java [13], making it the largest consumer of food in Indonesia. Within West Java, Bogor Regency has the largest population, totaling 5,473,476 people.

### Sample size and sampling technique

The sample size was determined following the Indonesian National Standard (SNI) 19–3964-1994 for the collection and measurement of samples of urban waste generation and composition. The sample size considered the population size, number of households, and average number of household members in the two selected sub-districts (Cibinong and Sukajaya Sub-district). Sample size formulation:

$$\begin{array}{cc}{\varvec{K}}=\frac{{\varvec{S}}}{{\varvec{N}}}& \mathbf{S}={\mathbf{C}}_{\mathbf{d}}\surd {\varvec{P}}\mathbf{s}\end{array}$$

Note: K = Number of household samples (household); S = Number of samples (people); N = Number of family members C_d_ = Housing coefficient (metropolitan and big cities = 1); Ps = Population (people).

The total population in Sukajaya Sub-district is 66,922 people, and in Cibinong Sub-district, it is 363,424 people [13], with an average of 4.03 people per household in Bogor Regency, [14]. Therefore, the total sample size for this study was 215 households, which were collected from 24 neighborhood associations or the smallest administrative units in Bogor Regency (*Rukun Tetangga*/RT). Multistage random sampling was conducted to collect data from the two sub-districts and 24 RTs, which were selected from a total of 102 RTs in the study location.

### Household food waste measurement

Household food waste was measured using two methods: Waste Composition Analysis (WCA) for solid FW (food) and a diary for liquid FW (drink). The WCA method was used following the SNI 19–3964-1994 to collect and measure samples of urban waste generation and composition. FW was collected for eight consecutive days for each household, with FW from the home trash bin being sorted and weighed daily according to the food type. FW collection was carried out every morning until the afternoon by waste collectors. Each household was supplied with 8 pre-coded plastic trash bags.

For liquid waste, measurements were conducted using a diary method for seven consecutive days, following The Waste and Resources Action Program (WRAP) guidelines for measuring household food and drink waste disposed of down the drain [15]. The recording completed independently by one of the family members at home who is responsible for food preparation (typically the housewife). The diary form and recording guidelines were provided one day in advance, with recording procedures directly explained by the study coordinators to participants (housewives). Diaries were then collected on the final day of food waste collection.

Total FW is the sum of food and drink waste and is divided into two types: edible and inedible FW. Edible FW is food still fit for consumption, such as meat cuts, bread crumbs, apples, etc. Meanwhile, inedible FW is food that is not fit for consumption or is not consumed under normal conditions, such as fish bones, eggshells, fruit peels, etc.

### Food security measurement

Household food security was assessed by examining the dimension of food access, measured using the Food Insecurity Experience Scale (FIES) questionnaire. Additionally, household income and the proportion of food expenditure were considered as confounding factors in this study. Household income (per capita per month) was categorized based on its data distribution: quartile 1 (< IDR 400,000), quartile 2 (IDR 400,000 to less than IDR 875,000), quartile 3 (IDR 875,000 to less than IDR 1,375,000), and quartile 4 (> IDR 1,375,000).

The FIES is an instrument for measuring food security, which serves as a proxy for food access and was developed by the FAO [16]. Data was collected using the FIES with a 30-day reference period, assessing household food insecurity experienced in the past 30 days. The FIES consists of three domains that define the food insecurity construct: uncertainty or worry about food sufficiency, inadequate food quality, and inadequate food quantity. These domains were described using eight questions related to the experience of hunger or food insecurity felt by family members due to a lack of money or other resources.

The eight questions were framed with a Yes or No response and addressed the following experiences: (1) worrying about not having enough food; (2) Not being able to eat healthy and nutritious food; (3) eating only a few types of food; (4) not eating breakfast, lunch, or dinner (skipping meals); (5) eating less than they should; (6) running out of food; (7) feeling hungry but not eating; and (8) not eating all day.

There were three levels of food insecurity severity: mild, moderate, and severe. Households that experienced uncertainty or worry about food sufficiency and inadequate food quality were classified as mildly food insecure (answered "yes" to questions 1–3). Households experiencing inadequate food quantity were classified as moderately food insecure (answered yes to questions 4–6) and finally households experiencing hunger were classified as severely food insecure (answered yes to questions 7–8) [16]. The prevalence of food insecurity was determined based on moderate and severe food insecurity.

Data collection on household characteristics (household size, expenditure, and the husband’s and wife’s occupation and education level) was conducted using a structured questionnaire. Interviews were conducted in participants' homes by trained interviewers who had graduated from nutrition and public health programs. The training of interviewers was carried out prior to the data collection.

### Statistical analysis

The association between household food security status and household characteristics was analyzed using the Maximum Likelihood Ratio Chi-Square test due to the unmet expected cell requirements. Spearman's Rank Test was employed to examine the association between household income, food expenditure proportion, and household food waste. Additionally, the association between household food security status and food waste was evaluated using Kendall Tau-b. Ordinal logistic regression was conducted to further explore the association between household food security status and food waste while adjusting for household characteristics, income, and the proportion of food expenditure. The threshold for statistical significance was set at α = 0.05. All inferential analyses were performed using IBM SPSS version 21.

## Results

In general, most households in this study had small families, with both spouses having a low level of education (≤ 9 years). The husbands were mostly entrepreneurs, laborers, and private employees, while the wives were mostly housewives. Furthermore, most of these families had a low proportion of food expenditure. The average household expenditure per capita per month was IDR 1,039,536, which is equivalent to USD 66 (food expenditure 44.6%; non-food expenditure 55.4%) (Table 1). The three largest contributors to food expenditure were animal products, cereals, tubers, and prepared food and beverages (Data not shown in the table). Table 1 shows that the prevalence of household food insecurity was 18.6%, with 11.2% classified as moderate and 7.4% as severe food insecurity. On the other hand, the average household FW was 77 kg/cap/year, with 37.7% being edible and 62.3% inedible FW. Fruits, cereals, tubers, and their derivatives, and vegetables were the largest contributors to total FW. The highest contributors to edible FW were cereals, tubers, and their derivatives (especially rice), while inedible FW was primarily fruits (especially bananas).

**Table 1:** Distribution of the study households according to social characteristics, food waste, and food security

Characteristics	n	%
**Household size (people)**		
Small (≤ 4 people)	152	70.7
Medium (5–6 people)	54	25.1
Large > 6 people)	9	4.2
**Husband’s education level**		
< 9 years	108	52.9
9–12 years	80	39.2
> 12 years	16	7.9
**Wife’s education level**		
<9 years	142	66.1
9–12 years	56	26.0
> 12 years	17	7.9
**Husband’s occupation**		
Laborer	48	23.5
Entrepreneur	55	27.0
Private employee	54	26.5
Farmer	11	5.4
Other	36	17.6
**Wife’s occupation**		
Housewife	176	81.9
Entrepreneur	15	7.0
Other	24	11.1
**Household expenditure [Mean (%)]**		
Food (IDR/cap/month)	441,531 (44.6)	
Non-food (IDR/cap/month)	598,005 (55.4)	
Total (IDR/cap/month)	1,039,536 (100)	
**Household food expenditure proportion**		
Low (< 60%)	189	87.9
High (≥ 60%)	26	12.1
**Household food security status**		
Food secure / mild food insecurity	175	81.4
Moderate food insecurity	24	11.2
Severe food insecurity	16	7.4
**Household food waste [Mean (%)]**		
Edible (kg/cap/year)	29.0 (37.7)	
Inedible (kg/cap/year)	48.0 (62.3)	
Total (kg/cap/year)	77.0 (100)	

Table 2 shows that food-secure households typically have higher income and education levels for the wives, while households experiencing severe food insecurity tend to have larger families. Additionally, households with moderate and severe food insecurity had low incomes (in quartiles 1 and 2). The husbands' education levels were comparably distributed across all categories of food insecurity. In addition, most households had a low proportion of food expenditure regardless of their food security status.

**Table 2:** Association between household characteristics, food waste, and household food security status

Household characteristics	Food secure/ mild food insecurity (n(%))	Moderate food insecurity (n(%))	Severe food insecurity (n(%))	*P* value
**Household size**				
Small	124 (81.6)	21 (13.8)	7 (4.6)	0.041^a^
Medium	43 (79.6)	3 (5.6)	8 (14.8)	
Large	8 (88.9)	0 (0)	1 (11.1)	
**Husband’s education level**				
<9 years	86 (79.6)	16 (14.8)	6 (5.6)	0.339^a^
9–12 years	69 (86.3)	5 (6.3)	6 (7.5)	
> 12 years	12 (75)	2 (12.5)	2 (12.5)	
**Wife’s education level**				
< 9 years	109 (76.8)	22 (15.5)	11 (7.7)	0.031^a^
9–12 years	51 (91.1)	1 (1.8)	4 (7.1)	
> 12 years	15 (88.2)	1 (5.9)	1 (5.9)	
**Household income**				
Quartile 1	31 (63.3)	10 (20.4)	8 (16.3)	0.008^a^
Quartile 2	46 (79.3)	7 (12.1)	5 (8.6)	
Quartile 3	49 (90.7)	4 (7.4)	1 (1.9)	
Quartile 4	49 (90.7)	3 (5.6)	2 (3.7)	
**Household food expenditure proportion**				
Low (< 60%)	155 (82)	20 (10.6)	14 (7.4)	0.777^a^
High (≥ 60%)	20 (76.9)	4 (15.4)	2 (7.7)	
**Food waste (mean kg/cap/year)**				
Edible	26.4	15.2	16.2	0.044^b^
Inedible	47.3	36.2	31	0.103^b^
Total	73.7	51.4	47.1	0.038^b^

^a^*P* value for Likelihood Ratio

^b^*P* value for Kendall Tau-b

Household FW decreased with the increasing severity of household food insecurity. Edible FW (*p* = 0.044; *r* = -0.110) and total FW (*p* = 0.038; *r* = -0.114) were negatively correlated with household food insecurity, whereas no significant correlation was observed between inedible FW and household food insecurity (*p* = 0.103; *r* =—0.089).

**Fig. 1:**
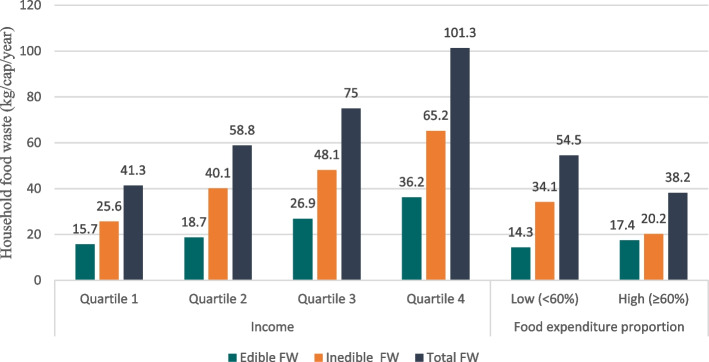
Household food waste (kg/cap/year) based on household income and proportion of food expenditure with household food waste

The average household FW increased as food access improved, as measured by income, proportion of food expenditure, and the FIES questionnaire. Households with high incomes, a low proportion of food expenditure, and food-secure households had higher FW than their counterparts. Figure 1 shows that household income was positively correlated with edible FW (*p* < 0.001, *r* = 0.288), inedible FW (*p* < 0.001, *r* = 0.379), and total FW (*p* < 0.001, *r* = 0.406). However, households with a low proportion of food expenditure generated more inedible food waste (*p* = 0.004, *r* = -0.197) and total food waste (*p* = 0.013, *r* = -0.170) than those with a high proportion. In contrast, edible FW was not significantly correlated with the proportion of household food expenditures.

The Kendall Tau-b analysis revealed an association between household food insecurity status and edible and total FW. As the severity of food insecurity increased, the generated FW decreased accordingly (see Table 2). After adjusting for household income, the proportion of food expenditure, and the education levels of both spouses, the association between food waste and food insecurity status became statistically insignificant (*p* > 0.05). Households in the lowest income quartile were 6.1 times more likely to experience food insecurity compared to those in the highest quartile (Table 3).

**Table 3:** Association between household food security status and food waste after adjusting for potential confounding factors

Variables	Estimate	*P*	OR (95% CI)
**Household size**			
Small	0.507	0.632	1.7 (-1.57 to 2.58)
Medium	0.548	0.614	1.7 (-1.59 to 2.68)
Large	Ref		
**Husband’s education level**			
< 9 years	-2.231	0.018	0.18 (-4.08 to -0.38)
9–12 years	-1.739	0.047	0.047 (-3.45 to -0.02)
> 12 years	Ref		
**Wife’s education level**			
< 9 years	1.282	0.238	0.236 (-0.85 to 3.14)
9–12 years	0.487	0.659	0.659 (-1.68 to 2.65)
> 12 years	Ref		
**Household income**			
Quartile 1	1.807	0.008	6.1 (0.48 to 3.14)
Quartile 2	0.993	0.129	2.7 (-0.29 to 2.28)
Quartile 3	0.139	0.848	1.1 (-1.29 to 1.56)
Quartile 4	Ref		
**Household food expenditure proportion**			
Low (< 60%)	-0.121	0.843	0.9 (-1.32 to 1.08)
High (≥ 60%)	Ref		
**Food waste (mean kg/cap/year)**			
Total	-0.005	0.408	1.0 (-0.02 to 0.01)
Edible	-0.003	0.862	1.0 (-0.03 to 0.03)
Inedible	Ref		

**Fig. 2:**
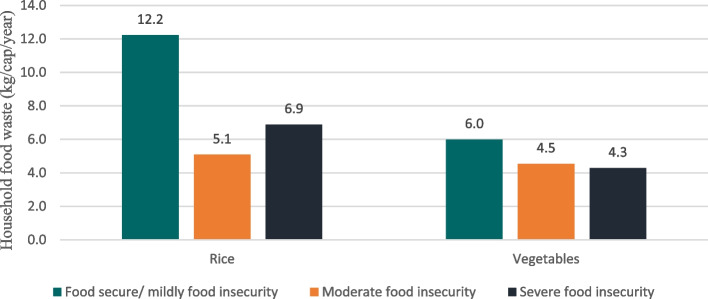
Discarded rice and vegetables (kg/cap/year) based on household food insecurity status

The largest contributors to edible FW were cereals, tubers, and their derivatives (especially rice), as well as vegetables such as carrots, mustard greens, cabbage, and cucumber. Food-secure or mildly food-insecure households (12.2 kg/cap/year) discarded more rice than moderately food-insecure (5.1 kg/cap/year) and severely food-insecure households (6.9 kg/cap/year). Similarly, the amount of discarded vegetables was slightly higher for food-secure or mildly food-insecure households (6 kg/cap/year) than for those with moderate (4.5 kg/cap/year) and severe food insecurity (4.3 kg/cap/year) (Fig. 2).

## Discussion

The prevalence of household food insecurity in this study (18.6%) was higher than both the national (11.9%) [17] and Southeast Asian (16.8%) prevalence but lower than the global (29.6%) and Asian (24.2%) prevalence [18]. Socioeconomic factors, such as income, household size, employment status, and education level, can influence household food security. Income affects a household's ability to purchase food, and a larger household size leads to increased food expenditure. The age and sex of household members can also affect their food intake and nutritional requirements. As a result, food-insecure households are at risk of malnutrition [19].

Low-income households were six times more likely to experience food insecurity than those with high incomes. A low household income can lead to low purchasing power, which in turn can reduce family food consumption. In addition, families with low-income levels tend to consume a less diverse or lower-quality diet compared to high-income families [20].

The average household FW in this study (77 kg/cap/year) was similar to the average household FW in upper-middle-income countries (76 kg/cap/year) and high-income countries (79 kg/cap/year [1]. This finding suggests that household FW is a significant issue for all income groups worldwide.

This study found a negative association between food waste and household food security (*p* < 0.05). FW decreases as the severity of food insecurity increases. Food-secure households with higher incomes tend to generate more FW than their counterparts. After adjusting for household income, the proportion of food expenditure, and the education levels of both spouses, the association became insignificant (*p* > 0.05). This reveals that household income plays a more prominent role in determining household food waste than household food security status.

This study aligns with research conducted in China, Australia, and Lebanon, which showed that higher-income families produce more food waste [2, 21, 22]. Families with higher incomes frequently dine out, resulting in more food waste at home [22]. High-income families tend to have better dietary quality, which leads to a greater amount of FW. Individuals with higher diet quality are more likely to consume a greater quantity of vegetables and fruits, leading to a higher amount of FW [23]. Proper storage at the appropriate temperature is necessary to maintain the quality of nutrient-dense foods, such as vegetables, fruits, and animal products [8].

This study revealed that households with lower incomes and higher food expenditures had smaller amounts of inedible FW, particularly fruits and their derivatives. Conversely, households with more severe food insecurity had smaller amounts of edible FW, particularly rice. Food access can influence FW behavior such as meal planning, purchasing, cooking habits, and eating habits. These habits, in turn, can affect the amount of FW produced by households. Eating habits, such as dining out, food preferences, and reluctance to eat leftovers, can also contribute to household FW [2, 24, 25].

Our results show a similar trend to the USA, where food-insecure households produce less FW than food-secure ones. However, after adjusting for household income in this study the correlation was not statistically significant. This contrasts with findings from the USA, which showed a significant correlation between FW generation and the severity of household food insecurity [11]. A study in Iran suggested that reducing food waste could positively impact household food security [10]. Conversely, research from Saudi Arabia found that food-insecure households generated more FW than food-secure ones [9].

This study suggests that increasing income is an effective strategy for addressing food insecurity. However, higher income must be accompanied by increased awareness to reduce food waste, especially edible FW like rice and vegetables. Therefore, preventing and reducing household FW is crucial, particularly among families with good food access.

### Study limitations

This study used a cross-sectional design, so it cannot establish a causal relationship between FW and food security at the household level. However, it is the first study in Southeast Asia to examine the correlation between directly measured food waste and food insecurity at the household level. Various methods were used to measure the FW. This study measured household FW using the WCA and a diary method. The WCA method has a high level of accuracy due to direct weighing and waste sorting by type and composition, which eliminates the possibility of under-reporting [26]. However, this study only calculated the solid food waste from the bins and did not account for other channels of food waste, such as animal feed, compost, etc.

## Conclusion

This study found no significant association between household food security status and food waste. Household income plays a crucial role in determining the amount of FW generated by the household. Higher-income households tend to be food secure and have more food waste. In contrast, low-income households are more likely to experience food insecurity and waste less food. Further studies are needed to establish a causal relationship between FW and food security at the household level. Prevention and reduction of household FW are crucial, especially in families with good access to food. Strategies to reduce FW should focus on the largest contributors to edible FW such as rice and vegetables.

## Data Availability

Data are available upon reasonable request to the authors.

## Abbreviations

FW: Food waste
SNI: Indonesian National Standard
WRAP: The Waste and Resources Action Program
